# Correction: Remdesivir-bisPropionate, a better derivative of remdesivir against SARS-CoV-2: Comparison of *in vitro* and *in vivo* PK/PD Study as well as its therapeutic potential

**DOI:** 10.1371/journal.pone.0359020

**Published:** 2026-09-22

**Authors:** Ashok Chakraborty, Anil Diwan, Vijetha Chiniga, Vinod Arora, Yogesh Thakur, Jayant Tatake, Rajesh Pandey, Preetam Holkar, Neelam Holkar

In III: In Vivo study for Therapeutic potential of RDV-bP against SARS virus, toxicity and stability in vivo subsection of the Materials and Methods, the phrase “Route of Administration (Table 1)” should have been removed.

In III: In Vivo study for Therapeutic potential of RDV-bP against SARS virus, toxicity and stability in vivo subsection of the Materials and Methods, there is an error in the first sentence of the Sublingual paragraph of the bulleted point Administration of test articles. The correct sentence is: NV − −387, NV–387-RDV-bP-and placebo for the gummy preparations were administered as a paste and painting the inner cavity of mouth by the use of a tuberculin syringe with no needle as per schedule and quantities in Table 1.

There is a labelling error in Fig 6. Please see the correct Fig 6 here.

**Fig 6:**
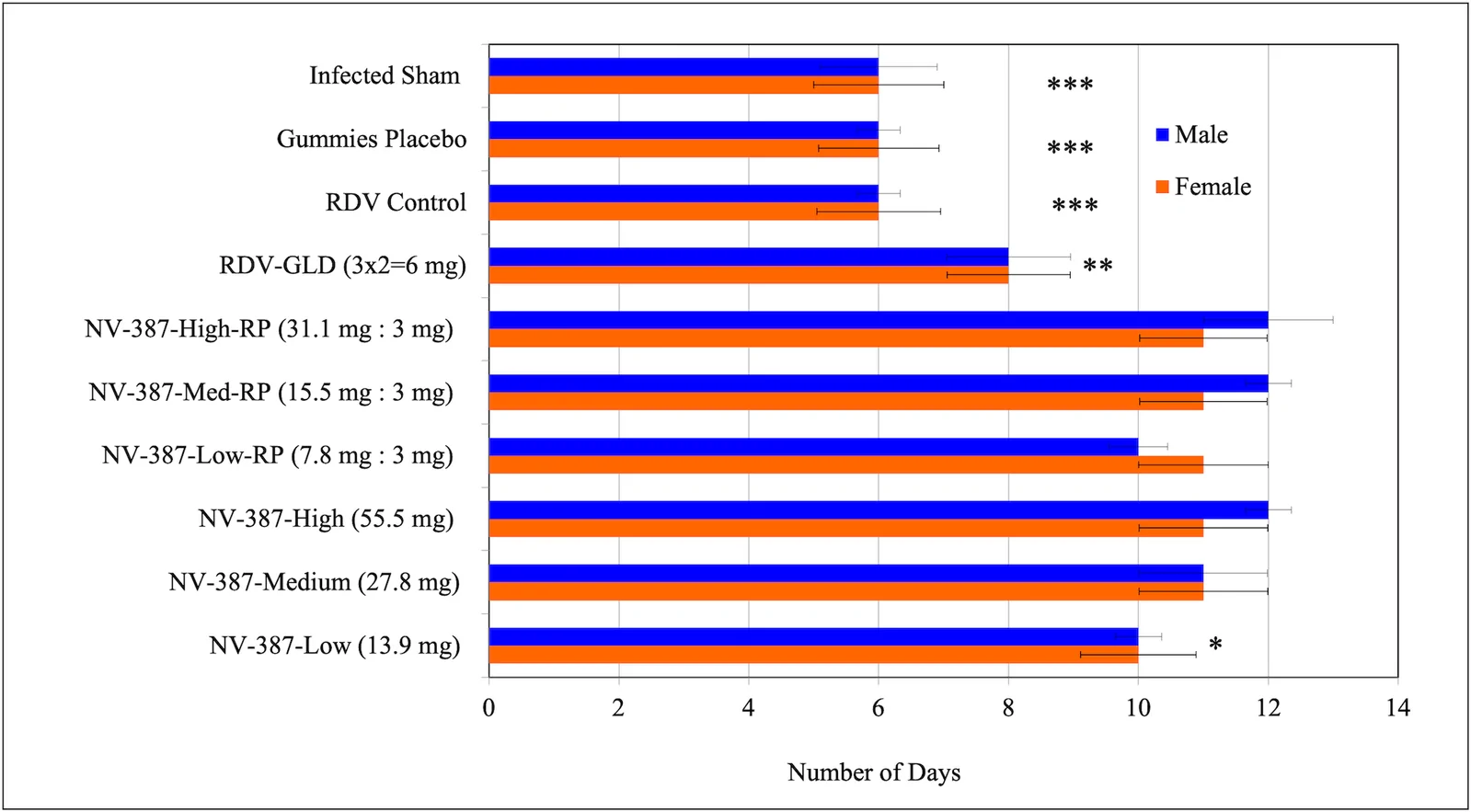
Antiviral Efficacy of RDV-bP on NL63-Infected Rats: Survival of Male and Female rats infected with NL-63 SARS virus and treated with NV387, NV387-RDV, NV387-RDV-bP and RDV-SBECD (GLD). Values are the mean ± SD from 3 experiments. Statistical analyses were done from two independent experiments done in duplicate. *: p < 0.5; **: p < 0.1; ***: p < 0.05.
