# Supplementary material for: Remdesivir-bisPropionate, a better derivative of remdesivir against SARS-CoV-2: Comparison of in vitro and in vivo PK/PD Study as well as its therapeutic potential
Source: PLoS One. 2026 Feb 20;21(2):e0324811. doi: 10.1371/journal.pone.0324811 (PMC12923118; doi:10.1371/journal.pone.0324811)
Supplement: Attachment — Submitted filename: Review Report PONE-D-24-39848.doc [file pone.0324811.s001.doc]

**PLOS ONE**

Title: **Remdesivir-bisPropionate, a better Derivative of Remdesivir against SARS-CoV-2: Comparison of *in vitro* and *in vivo* PK/PD Study as well as its Therapeutic Potential**

Manuscript # PONE-D-24-39848

Review Report:

1. The abstract should be in a single paragraph with continuous form without breaking the sentences. Need to change the format.
2. The introduction part is not according to the journal style. So many sub headings in without objectives of the research work.
3. The materials and methods section, the authors just cut and paste of the student’s thesis, without following the PLOS ONE authors guideline.
4. The results and discussion part needs to rewritten.
5. Tables and figures are necessary. But the tables format needs to be changed as well as the clarity of the figures need to be improved.

The manuscript in this present is not sufficient for the PLOS ONE journal. So it may be rejected.
